# Correction: ‘Mechanistic analysis of the search behaviour of *Caenorhabditis elegans*’ (2014), by Salvador *et al.*

**DOI:** 10.1098/rsif.2025.0557

**Published:** 2025-07-23

**Authors:** Liliana C M Salvador, Frederic Bartumeus, Simon A Levin, William S Ryu

**Affiliations:** ^1^ Animal and Comparative Biomedical Sciences, University of Arizona, Tucson, AZ, USA; ^2^ Centre d'Estudis Avançats de Blanes, Acces Cala Sant Francesc 14, Blanes, Girona, Spain; ^3^ Department of Ecology and Evolutionary Biology, Princeton University, Princeton, NJ USA; ^4^ Department of Physics, University of Toronto, 60 St George St., Toronto, Ontario, Canada

*J. R. Soc. Interface* **11**: 20131092 (Published online 06 March 2014). (http://doi.org/10.1098/rsif.2013.1092)

The URL to the dataset used in this paper has changed; the original paper has been updated accordingly.

**Data accessibility**

The *Caenorhabditis elegans* image data are stored in the following repository: https://doi.org/10.6084/m9.figshare.28928159.

